# Recurrent laryngeal nerve injury after thyroidectomy: A national study from Saudi Arabia

**DOI:** 10.15537/smj.2023.44.1.20220710

**Published:** 2023-01

**Authors:** Saad M. Alqahtani, Hanan R. Al-sohabi, Musaed F. Rayzah, Amani S. Alatawi, Areej A. AlFattani, Yousef S. Alalawi

**Affiliations:** *From the Department of Surgery (Alqahtani, Rayzah), College of Medicine, Majmaah University, Al-Majmaah, from the Department of Surgery (Al-sohabi, Alalawi), King Salman Armed Forces Hospital Northwestern Region, from the Department of Surgery (Alatawi), King Fahad Specialist Hospital, Tabuk, and from the Department of Biostatistics, Epidemiology, and Scientific Computing (AlFattani), King Faisal Specialist Hospital and Research Centre, Riyadh, Kingdom of Saudi Arabia*

**Keywords:** complications of thyroidectomy, recurrent laryngeal nerve injury, thyroidectomy

## Abstract

**Objectives:**

To determine the incidence and possible risk factors of recurrent laryngeal nerve injury, to provide a relevant literature review of studies from other centers in Saudi Arabia, and to present basic statistical data for future studies in our local community.

**Methods:**

A retrospective study enrolled patients who were surgically treated for thyroid disease between January 2015 and December 2021. For concerns during the procedure, direct laryngoscopy was carried out before extubation to assess the vocal cords. Similarly, indirect laryngoscopy was carried out for patients who developed postoperative voice changes. All patients were evaluated clinically 2-3 weeks after surgery. Nerve monitors were not used in either case.

**Results:**

The study examined 437 participants: 361 (82.6%) female and 76 (17.4%) male individuals. The incidence of recurrent laryngeal nerve injury was 1.1%. The demographic characteristics, pathology (benign vs. malignant), and extent of thyroidectomy were not significantly associated with the risk of recurrent laryngeal nerve injury.

**Conclusion:**

A recurrent laryngeal nerve injury is a serious complication, and further studies are required to determine the safest techniques for thyroidectomy. However, centralization of thyroid surgery in high-volume centers might reduce this risk.

**T**hyroid disease is the most common endocrine disorder worldwide. Its treatment can be either through pharmaceutical or surgical interventions, with thyroid surgery being one of the most common procedures carried out globally.^1^ At present, thyroid surgery is a relatively safe procedure. This could be attributed to advances in anesthesia, better surgical equipments, improved operative techniques and antisepsis, and a better understanding of thyroid gland anatomy and physiology. Nonetheless, like other surgical procedures, thyroidectomy can potentially result in postoperative complications.^1^

Hypoparathyroidism, recurrent laryngeal nerve (RLN) injury, hematoma, loss of high-pitched voice, seroma, thoracic duct injury, and wound infection are well-known post-thyroidectomy complications. Of these, the first 2 are the most frequently encountered.^1^ The most feared complication of thyroid surgery is RLN injury, which has a negative impact on a patient’s quality of life.^1^ Injury may be either transient or permanent, with Transient referring to an injury that has a complete recovery within 4-6 weeks or, in some reports, up to a year. Otherwise, nonfunctions or dysfunctions lasting more than 12 months are considered permanent.^2^

Factors such as extent of surgery, pathology type (such as Graves’ disease and cancer), gland volume, retrosternal extension, neck dissection, and re-operative procedure reportedly influence the risk of RLN injury.^3,4^

Unlike previous publications from Saudi Arabia, this study examined post-thyroidectomy RLN injury in relation to age, gender, type of pathology, and extent of surgery. Furthermore, this study aimed to estimate the risk factors and prevalence of RLN injury, to provide a relevant literature review of studies from other centers in Saudi Arabia, and to present basic statistical data for future studies in our local community.

## Methods

This retrospective study was carried out in King Salman Armed Forces Hospital, Northwestern Region, Tabuk, Saudi Arabia, between January 2015 and December 2021. We enrolled 437 male and female patients who underwent thyroid surgeries. Patients with symptomatic preoperative RLN palsy were excluded.

Data were retrieved from the patients’ medical records. Intraoperative vocal cord assessment was carried out before extubation via direct laryngoscopy if there was a concern during the procedure. Moreover, vocal cords were assessed via indirect laryngoscopy in patients who developed postoperative voice changes. Additionally, all patients were evaluated clinically 2-3 weeks after surgery. Nerve monitors were not used in either case.

We routinely visualize the RLN during surgery at our institution. Routine preoperative vocal cord assessment was not carried out at our center unless there were compressive symptoms, such as voice changes or prior redo surgery.

The records included demographic information (age and gender), final pathology (benign vs. malignant), and surgical extent (hemi, subtotal, completion, or total thyroidectomies).

The study was approved by the Review Board of King Salman Armed Forces Hospital, Northwestern Region, Tabuk, Saudi Arabia (approval number KSAFH-REC-2017166) and was carried out according to principles set forth in the Declaration of Helsinki. The informed consent was not required due to the retrospective design of the study.

### Statistical analysis

All statistical analyses were carried out using the Statistical Packages for the Soccial Sciences, version 22.0 (IBM Corp., Armonk, NY, USA). Data are presented as frequencies and percentages for quantitative variables and as means and standard deviations (SD) for continuous variables. To determine the relationship between the RLN injury and extent of thyroidectomy and other factors we used Chi-square independent test or Fisher’s exact test were appropriate. A *p*-value of <0.05 was considered significant.

## Results

This study included 437 patients who underwent thyroid surgeries. The majority of patients were female (n=361, 82.6%), with men accounting for only 17.4%. The ages ranged between 13-95 years, with a mean of 40.5±13 years. Benign pathologies were observed in 239 (54.7%) patients, while malignant pathology was observed in 198 (45.3%) patients. Total thyroidectomy accounted for the most commonly carried out procedure (n=256, 58.6%). Right hemithyroidectomies was carried out in 82 (18.8%) patients and left hemithyroidectomies was carried out in 63 patients (14.4%). Completion thyroidectomy was carried out in 29 (6.6%) patients of the entire group, whereas the remaining patients underwent subtotal thyroidectomy (n=7, 1.6%). Table 1 summarizes the characteristics of patients with RLN injury, including age, type of surgery, pathology type, and other features of the thyroid gland.

**Table 1:** - Characteristics of patients with recurrent laryngeal nerve injury.

Patient no.	Age (years)	Pathology	Type of pathology	Procedure	Type of injury	Side	Neck dissection	Other features
1	63	Malignant	Papillary thyroid cancer	Total thyroidectomy	Temporary	Unilateral	Yes	-Retrosternal extension-Extrathyroidal extension
2	28	Benign	Diffuse toxic goiter	Total thyroidectomy	Temporary	Unilateral	No	-
3	39	Benign	Multinodular goiter	Total thyroidectomy	Temporary	Unilateral	No	Retrosternal extension
4	43	Benign	Hashimoto’s thyroiditis	Right-hemi thyroidectomy	Temporary	Unilateral	No	Adhesions
5	41	Benign	Multinodular goiter with cystic degeneration	Total thyroidectomy	Permanent	Bilateral	No	-Huge left lobe-Adhesions and fibrosis

Our results showed that only 5 (1.1%) female patients developed RLN injury. Four of these patients experienced only transient unilateral injury and recovered completely. The fifth patient had permanent bilateral RLN injury and did not require tracheostomy. The final histopathological results showed benign pathology in 4 patients, while malignancy was observed in one.

There was no statistically significant relationship between age, gender, final pathological diagnosis (benign vs. malignant), the extent of thyroidectomy (hemi, subtotal, completion, or total thyroidectomies), and the risk of RLN injury (Table 2).

**Table 2:** - Associations of patient’s demographics, final pathology, and extent of thyroidectomy with the risk of recurrent laryngeal nerve.

Variables	RLN injury		
	No (n=432)	Yes (n=5)	*P*-values*
Age (years), mean±SD	41.2±19.1	42.8±12.6	0.84
***Gender***			
Female	356 (82.4)	5 (100)0	0.59
Male	76 (17.6)	0 (0.0)	
***Final pathology***			
Benign	235 (54.4)	4 (80.0)	0.38
Malignant	197 (45.6)	1 (20.0)	
***Extent of thyroidectomy***			
Total thyroidectomy	252 (58.3)	4 (80.0)	0.81
Right hemi-thyroidectomy	81 (18.8)	1 (20.0)	
Left hemi-thyroidectomy	63 (14.6)	0 (0.0)	
Sub-total thyroidectomy	7 (1.6)	0 (0.0)	
Completion thyroidectomy	29 (6.7)	0 (0.0)	

Values are presented as numbers and precentages (%).

* Significant at a *p*-value of <0.05. RLN: recurrent laryngeal nerve, SD: standard deviation

## Discussion

In the present study, RLN injury after thyroid surgery was observed in only 5 (1.1%) patients, which is in line with previously published studies.^2,5^ Table 3 depicts the incidence among different centers in Saudi Arabia.^6-16^ Although all 5 patients were female, our data revealed no statistically significant relation between gender, age, and the risk of nerve injury, which is also consistent with previous studies.^1,9,12^

**Table 3:** - Prevalence of recurrent laryngeal nerve injury among different cities in Saudi Arabia.

Studies	Years	Cities	RLN injuries
Bawa et al^6^	2021	Bisha	4 (1.9)
Alghamdi et al^7^	2021	Makkah	5 (1.9)
Al-Essa et al^8^	2021	Riyadh	6 (4.9)
Al-shareef et al^9^	2020	Makkah	9 (7.0)
Al-kaff et al^10^	2020	Makkah	4 (1.3)
Qobty et al^11^	2020	Aseer	37 (24.7)
Al-Hakami et al^12^	2019	Jeddah	17 (3.7)
Al-Harbi et al^13^	2018	Jazan	18 (5.6)
Al-Amri^14^	2014	Dammam	10 (7.35)
Alshahrani et al^15^	2013	Riyadh	0(0.0)
Zakaria et al^16^	2010	Al-Khobar	14 (4.1)
Present study	2022	Tabuk	5 (1.1)

Values are presented as numbers and precentages (%). RLN: recurrent laryngeal nerve

Injuries can be transient or permanent, unilateral, or bilateral. The incidence of transient injuries have been reported to be 4-7% and permanent injuries have been reported to be 1-4%.^5^ However, another study found that the incidence of transient injuries ranged from 1-30%, while that of permanent injuries ranged from 0.5-5%.^2^ According to Oertli et al,^17^ the risk of transient injury was 3.5-5.2% and 0.5-2.4% of permanent injury. In contrast, Reeve et al^18^ emphasized that the risk of injury was less than 1%. In our study, 4 (0.9%) patients had transient injuries, while one (0.2%) had a permanent injury.

The most common clinical manifestations of unilateral RLN injury are voice weakness and hoarseness. Conversely, dyspnea and glottal obstruction are common in bilateral RLN injury.^16^ All our patients with RLN injury developed hoarseness. Four of the patients subsequently recovered and regained their normal vocal qualities. The fifth patient had permanent bilateral RLN injury. Despite that, she did not require a tracheostomy.

There are several mechanisms of RLN injury. These include ischemia, traction, stretching, entrapment, thermal injury, contusion, hematoma, compression, transection, irritation without actual damage, and endotracheal cuff compression.^5^ In the latter (due to vocal fold edema), the voice changes usually resolve in a few days. In rare occasions, RLN transection may occur or be required during thyroid surgeries. Therefore, re-anastomosis should be carried out with the aim to restore vocalis muscular tone. In contrast, transient RLN dysfunction could develop if the RLN is stretched during surgery and in such cases, calcium channel blockers are helpful in recovery.^19^

Graves’ disease, huge multinodular goiter, thyroid malignancy, the extent of surgery, central lymph node dissection, redo surgeries, and the inexperience of surgeons are all precipitating factors for RLN injury.^1,2,16^ In our study, patient one had a malignant pathology with retrosternal and extrathyroidal extensions and required central neck dissection. Patient 2 had Graves’ disease, while patient 3 had retrosternal extension. Additionally, patient 4 had only adhesions, and lastly, patient 5 had a large left lobe with adhesions and fibrosis.

Compared with other conservative procedures, total thyroidectomy has been reported to have a higher risk of nerve injury.^6,16^ However, our data showed no significant relationship between the extent of thyroidectomy and the risk of RLN injury. Karamanakos et al^1^ also found no relationship between the extent of thyroidectomy and the risk of RLN injury in their univariate analysis only. However, their multivariate analysis identified pathology type (malignancy, recurrent goiter, and thyroiditis) as an independent risk factor for transient RLN injury, which contradicts our findings.^1^ In addition to these factors, anatomical distortions and variations may increase the risk of RLN injury.^20^

Although RLN injury cannot be totally prevented, careful intraoperative inspection and understanding of its anatomical course, along with its variations, should significantly reduce the risk.^2,18^ Anatomically, the left RLN has a straightforward course in the neck, whereas the right RLN follows a variable course. The right RLN is slightly more lateral than the left RLN. Additionally, it is important to know the different relationships between the inferior thyroid artery and the RLN. The artery may be in front or behind the RLN. It may also pass between the nerve and its branches or split before reaching the nerve.^20^ Some experienced surgeons do not routinely expose the nerve but are still able to avoid RLN injury. However, visualization is still recommended, at least during a portion of the procedure.^18^ This is also a routine practice at our institution. According to Dralle et al,^21^ visual nerve identification is the gold standard strategy for thyroid surgery. Furthermore, in a meta-analysis, systematic nerve visualization was found to be the best step for avoiding RLN injury.^22^

There is still controversy regarding recurrent nerve dissection and whether it increases the risk of RLN injury. Several studies have concluded that recurrent nerve dissection was unnecessary in subtotal thyroidectomy; however, it is beneficial in complicated cases, such as thyroid malignancy.^6,16^ Echternach et al^23^ found that recurrent nerve dissection did not increase the risk of both temporary and permanent RLN injuries. Surprisingly, they reported that the risk of permanent RLN injury was reduced, implying that recurrent nerve dissection is advised.^23,24^

The use of neuromonitoring is similarly controversial. Some authors have advocated for nerve monitoring to reduce the incidence of RLN injury, while others have found no statistically significant evidence to support its use.^16^ Furthermore, it did not aid in the anatomical dissection of the RLN.^2,16^ The risk of injury has been reported to be 3.1% in cases of nerve visualization; 3.5% when a nerve stimulator was used.^22^ Some authors have also argued that nerve monitoring should be used only in redo surgeries, huge goiters, malignant cases, and widespread metastatic lymph node invasion.^3^

Different treatment approaches for RLN injuries have been proposed, including voice therapy, vocal exercises, medialization, injection laryngoplasty, and arytenoid adduction.^2,25^ Tracheostomy is indicated in permanent bilateral RLN injuries associated with severe dyspnea.^3^

### Study’s strenght & limitations

The strength of our study lies in the literature review of the incidence of RLN injury at other centers in Saudi Arabia. Furthermore, unlike other previous publications from Saudi Arabia, this study examined post-thyroidectomy RLN injury in relation to age, gender, type of pathology, and extent of surgery. Nevertheless, it has some limitations, such as its retrospective nature, being single center study, small sample size, and low incidence of RLN injury. Additionally, post-operative vocal cord palsy cases that were not overtly symptomatic may be missed, resulting in under-estimation of exact incidence and selection bias.

In conclusion, RLN injury is a serious complication. Prospective multicenter studies are required to determine the safest thyroid surgery techniques and improve treatment plans. Additionally, to overcome or reduce the rate of RLN injury, all possible modifiable risk factors need to be determined. Furthermore, thyroidectomy should be carried out by skilled surgeons at high-volume hospitals. In addition, pre- and post-operative vocal cord evaluation should be considered in a future study to obtain an accurate incidence of vocal cord palsy.
